# Hugo™ Versus daVinci™ Robot-Assisted Radical Prostatectomy: 1-Year Propensity Score-Matched Comparison of Functional and Oncological Outcomes

**DOI:** 10.3390/jcm13226910

**Published:** 2024-11-16

**Authors:** Carlo Gandi, Filippo Marino, Angelo Totaro, Eros Scarciglia, Simona Presutti, Fabrizio Bellavia, Riccardo Bientinesi, Filippo Gavi, Francesco Rossi, Seyed Koosha Moosavi, Giuseppe Palermo, Marco Racioppi, Nicolò Lentini, Roberta Pastorino, Emilio Sacco

**Affiliations:** 1 Department of Urology, Fondazione Policlinico Universitario Agostino Gemelli IRCCS, 00168 Rome, Italy; carlogandi@gmail.com (C.G.); angelo.totaro@policlinicogemelli.it (A.T.); erosscarciglia@gmail.com (E.S.); simopresutti@hotmail.it (S.P.); bellaviafabrizio@gmail.com (F.B.); riccardo.bientinesi@policlinicogemelli.it (R.B.); filippogavimd@gmail.com (F.G.); dottorfrancescorossi@gmail.com (F.R.); moosavi.kooshamd@gmail.com (S.K.M.); giuseppe.palermo@policlinicogemelli.it (G.P.); marco.racioppi@policlinicogemelli.it (M.R.); 2 Department of Medicine and Translational Surgery, Università Cattolica del Sacro Cuore, 00168 Rome, Italy; emilio.sacco@unicatt.it; 3 Department of Life Sciences and Public Health, Section of Hygiene, Università Cattolica del Sacro Cuore, 00168 Rome, Italy; nicol.lentin@libero.it (N.L.); roberta.pastorino@unicatt.it (R.P.); 4 Department of Woman and Child Health and Public Health—Public Health Area, Fondazione Policlinico Universitario Agostino Gemelli IRCCS, 00168 Rome, Italy; 5 Department of Urology, Ospedale Isola Tiberina—Gemelli Isola, 00168 Rome, Italy

**Keywords:** RARP, robot-assisted radical prostatectomy, prostate cancer, Hugo RAS, daVinci, robotic surgery, comparative analysis, propensity score

## Abstract

**Background/Objectives**: A comprehensive comparison of intraoperative, oncological, and functional outcomes of RARP performed with different robotic surgical platforms is critically needed. Our aim is to compare the oncological and functional outcomes of RARP performed using the novel Hugo™ RAS system with those from the daVinci system, the reference standard, at a high-volume robotic center, with an extended follow-up period (one year). **Methods**: We analyzed the data of 400 patients undergoing RARP ± pelvic lymph node dissection between 2021 and 2023, using propensity score (PS) matching to correct for treatment selection bias. All procedures were performed by three surgeons with HugoTM RAS or daVinci. This analysis extends the follow-up period to 1 year, focusing on specific functional and oncological outcomes, building on our previous 3-month evaluation of perioperative outcomes. The primary outcome was the trifecta rate, defined as freedom from biochemical recurrence, continence, and erectile function recovery. Secondary outcomes included detailed assessments of oncological outcomes (PSA levels) and functional outcomes (continence and erectile function). **Results**: The propensity score-matched cohort included 99 matched pairs (198 patients), balanced for all covariates. No significant differences were found in trifecta rates between the two platforms at 1-year follow-up (Hugo: 25.25%, daVinci: 27.27%, *p* = 0.743). Both groups showed improved trifecta rates when considering only nerve-sparing procedures (Hugo: 36.84%, daVinci: 35.59%, *p* = 0.889). Continence rates were similar (Hugo: 87.9%, daVinci: 89.9%, *p* = 0.327), as were the undetectable PSA rates (Hugo: 92.9%, daVinci: 88.8%, *p* = 0.158). Also, the erectile function recovery rate did not differ significantly between the groups. **Conclusions**: This is the first study comparing 1-year functional and oncological outcomes of RARP performed with Hugo™ RAS and daVinci surgical robotic systems using PS matching. Functional and oncological outcomes of RARP were comparable between the two robotic platforms. These findings confirm that the transition to the Hugo™ platform does not compromise surgical proficiency or patient outcomes, even if further long-term studies are necessary to confirm these results.

## 1. Introduction

Robot-assisted radical prostatectomy (RARP) is the most adopted treatment for localized prostate cancer [1,2]. The widespread adoption of robotic surgery, particularly RARP, has revolutionized the approach to prostate cancer management over the past two decades [3]. This is due not only to the technical advantages that robotic platforms provide but also to the continuous improvements in patient outcomes, including reduced recovery times and lower complication rates.

Following the FDA (Food and Drug Administration) approval for prostate surgery in 2001, the daVinci Surgical System (Intuitive Inc., Sunnyvale, CA, USA) became the leading technology in urology over the next two decades. Its advantages were well-documented, benefiting both surgeons and patients [4].

However, its significantly higher costs have limited its widespread adoption as a standard practice, particularly in comparison to open and laparoscopic surgeries.

Since 2019, some of Intuitive’s patents have expired, allowing other manufacturers to introduce competitive robotic surgical platforms [5], which promise to reduce costs, the Achilles’ heel that detractors of robotic surgery have always pointed out.

Despite the initial hesitation due to high costs, the growing body of evidence demonstrating superior functional and oncological outcomes has strengthened the case for robotic surgery as the gold standard in many high-volume centers around the world. The continued development of alternative platforms, such as the Hugo™ RAS system, is an important step toward making robotic surgery more widely accessible.

The Hugo™ RAS (Medtronic, Minneapolis, MN, USA) multi-port modular robotic platform received CE (conformité européenne) approval for urologic surgeries in adults in October 2021. Since then, after several authors showed the feasibility of RARP performed with Hugo™ RAS with promising initial perioperative outcomes [6,7,8,9,10,11,12,13], this new platform is rapidly spreading within the new multiplatform robotic surgery landscape.

However, this rapid spread has created an urgent need for robust evidence of Hugo’s outcomes compared to those of the daVinci surgical system, which represents the reference standard for RARP, due to its extensive and long-term use that generated a vast amount of published evidence.

Several authors compared early post-operative outcomes of RARP performed with Hugo RAS and daVinci [14,15,16]. In our previous work, we already showed the results of a propensity score-matched comparative analysis of perioperative outcomes of RARP performed with Hugo RAS and daVinci [17].

Therefore, after comparing the perioperative outcomes, we aimed to compare 1-year oncological and functional outcomes of RARP performed with Hugo RAS and daVinci surgical system in the same population, at our high-volume tertiary referral robotic center, the first in Italy and second in Europe to introduce Hugo™ RAS for RARP in daily practice.

## 2. Material and Methods

### 2.1. Patient Population and Study Design

All the patients undergoing RARP at the Fondazione Policlinico Universitario A. Gemelli IRCCS (Rome, Italy) from September 2021 to September 2023 were screened for inclusion within this Institutional Review Board (IRB)-approved observational comparative study (ID 5119/2022). Patient data from a prospectively collected database were analyzed retrospectively. The inclusion criteria were (1) the procedure is performed using either the Hugo™ RAS or the daVinci Xi surgical systems; (2) the surgery must have been conducted by one of three experienced surgeons with over 500 RARP cases performed on the daVinci Xi platform and who had already achieved stable proficiency in achieving negative surgical margins [18]; (3) patients must have signed informed consent. Patients were excluded if they had missing data (such as the absence of magnetic resonance imaging information), if the procedure required conversion to open surgery, or if they had received preoperative hormone therapy. All patients were prescribed chronic tadalafil therapy.

As of March 2022, both the Hugo™ RAS and daVinci systems were in routine use at our center, with no preference given to either system. Recognizing a learning curve for the Hugo^TM^ RAS system, we excluded patients with high-risk disease requiring pelvic lymph node dissection, prior major abdominal surgery, previous trans-urethral resection of the prostate (TURP), or those who had undergone neoadjuvant hormone therapy, from the initial ten cases performed with Hugo^TM^ RAS. Following these first ten cases, patient inclusion in the Hugo^TM^ cohort commenced without any further selection criteria.

This study was conducted in accordance with the STROBE (Strengthening the Reporting of Observational Studies in Epidemiology) guidelines (in Table S1).

#### 2.1.1. Technical Overview of Hugo™ RAS and daVinci Systems

The Hugo™ RAS system consists of a system tower integrated with a Valleylab^®^ electrosurgical generator, up to four individual robotic arm carts, and an open console. Differing from the daVinci system’s closed console, isolating the surgeon to reduce external distractions during procedures, the Hugo™‘s RAS allows for a more open environment. This design encourages multitasking and easier communication among the surgical team. Moreover, Hugo™ offers an ergonomic console that improves surgeon posture, allowing greater freedom of head movement and flexibility in positioning during surgery [19,20]. Both systems offer 3D high-definition visualization but utilize different technologies. The daVinci system employs stereoscopic technology with two monitors—one for each eye—projecting images from an Intuitive™ 3D endoscope. In contrast, the Hugo™ system uses a passive 3D display, where the surgeon wears tracker-equipped glasses to view images captured by a Karl Storz 3D laparoscopic endoscope [21,22,23]. When it comes to instrument control, Hugo™ offers “pistol-like” hand controllers with a wrist rotation scaling factor of up to 2x and a rotation range of up to 520°, enhancing tasks like suturing in deep cavities. Both systems feature tremor filtering for more precise movements, but their pedal controls differ: daVinci allows instrument changes with a single pedal click, whereas Hugo™ requires holding the pedal down for 1.5 s [19,20,21,22]. Additionally, Hugo™ still lacks some advanced tools, such as Hem-O-Lok appliers and fast sealing devices, already available in the daVinci ecosystem [19,20]. Both the systems are equipped with AI-based video tools (My Intuitive for daVinci and Touch Surgery™ for Hugo™). However, Hugo™ goes a step further by using AI to automatically segment surgical videos into procedural steps. This could enhance experienced surgeons’ performance and simplify younger surgeons’ training by streamlining the learning process [21,22].

#### 2.1.2. Surgical Technique

All RARP procedures were carried out according to the widely recognized Montsouris technique [18,24], originally developed for laparoscopic prostatectomy and later adapted for robotic surgery. The specifics of the technique, including port placement and robotic docking configuration, have been outlined in detail in our previous publication [6]. The nerve-sparing technique was carried out in both groups according to individual oncological risk based on patient perioperative characteristics such as biopsy reports, MRI findings, and intraoperative findings. When using the Hugo^TM^ RAS system, the surgical instruments routinely employed included monopolar curved scissors, bipolar Maryland forceps, Cadière forceps, and large needle drivers [6]. On the daVinci platform, Prograsp forceps were used in place of Cadière forceps.

All surgeons participating in this study underwent comprehensive training, focusing on the Hugo™ RAS cart-docking system and open console controllers at ORSI Academy (Melle, Belgium). This training was team-based, incorporating not only the surgeons but also bedside assistants and scrub nurses from our institution. Additionally, almost every procedure was supported by a Medtronic specialist in our operating room, ensuring the system’s correct setup and smooth operation.

### 2.2. Measurements and Outcomes

Patients’ demographic and clinical data were obtained from the prospectively maintained database.

As a primary outcome, we chose a composite outcome that represented the achievement of an optimal post-operative status both functionally and oncologically.

Trifecta [25] and pentafecta [26] are the most widely adopted composite outcomes in studies evaluating long-term outcomes after radical prostatectomy. Trifecta combines the achievement of freedom from biochemical recurrence (BCR) along with recovery of continence and erectile function [25]. Pentafecta is an evolution of the concept of the trifecta, meaning the combination of five outcomes: no BCR, recovery of continence and erectile function, no positive surgical margins (PSM), and no post-operative complication [26].

In our case, PSM and post-operative complication rates were already evaluated and discussed in our previous work comparing perioperative outcomes of RARP performed with Hugo and daVinci. Thus, for the present study, we chose trifecta as the primary outcome in order to focus on long-term functional and oncological outcomes.

Secondary outcomes included oncological and functional outcomes taken separately. Recovery of continence was defined as social continence (no more than one pad per day) [27]. The 24-h pad use and 24-h pad weighing tests were also recorded. Recovery of erectile function was defined as a score ≥17 on the IIEF-5 questionnaire, which was administered to the patient both preoperatively and at a 12-month follow-up visit after surgery [28]. Additionally, the patient was asked to verbally indicate his erection status, choosing from the following options: present and sufficient for penetration, present but not sufficient for sexual intercourse, present but not sufficient for penetration, or absent.

## 3. Statistical Methods

Descriptive statistics were applied: continuous variables were summarized by the median along with the interquartile range (q1–q3), while categorical variables were expressed as both absolute frequencies and percentages. The independent t-test or Mann–Whitney U-test was used to compare continuous variables, depending on the distribution of the data [29]. Categorical variables were compared using either the Chi-squared test or Fisher’s exact test based on suitability.

A propensity score (PS) matching analysis was utilized to address potential confounding factors related to preoperative characteristics. PS-matching offers a method to estimate treatment effects by considering the conditional likelihood of treatment selection [30]. The propensity score, as a balancing metric, represents the likelihood of an individual receiving a specific treatment based on observed covariates. This method helps ensure that the baseline covariates distribution is similar between treated and untreated subjects, based on the propensity score, reducing bias in the comparison of treatment groups [30].

Propensity scores were calculated by a logistic regression model, incorporating covariates such as age, BMI, Charlson Comorbidity Index (CCI), previous abdominal surgery, preoperative PSA, prostate volume, and biopsy ISUP grade. A 1:1 PS-matching was performed using a greedy nearest-neighbour algorithm with a calliper width of 0.2 standard deviations of the logit of the propensity score without replacement. The covariate balance between groups was assessed by comparing baseline covariates and the cumulative distribution functions of the propensity scores of each matched pair [31].

Both p-values and standardized mean difference (SMD) were employed to compare variables across treatment groups [29]. An SMD value below 0.1 was considered indicative of adequate balance between the groups [32].

Logistic regression and ordinal regression models were developed to investigate the association between the use of robotic systems and both oncological and functional outcomes.

Before applying the proportional odds ordinal logistic regression model, the assumption of parallel lines was verified using the Brant test [33] to determine whether the effects of all independent variables remained consistent across the categories of the response variable.

Effect sizes were reported as odds ratios (ORs) with their corresponding 95% confidence intervals (CIs). A two-sided p-value of less than 0.05 was considered statistically significant.

“R”, version 4.2.0 (2022-04-22) for Windows, was used for all statistical analyses.

## 4. Results

### 4.1. Matching Procedure and Baseline Characteristics

**Figure 1:**
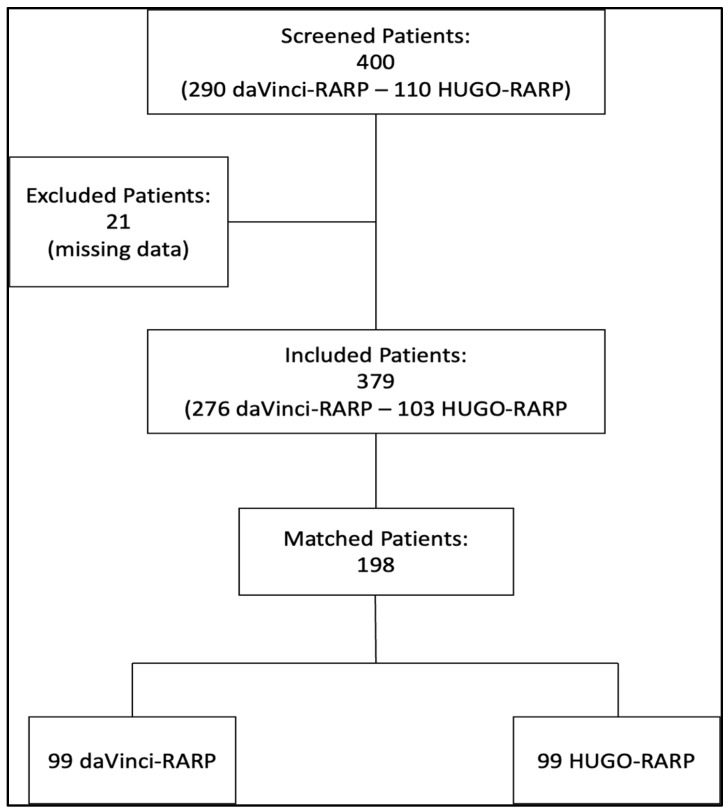
Study flowchart.

Out of 400 evaluated patients, 379 were deemed eligible after the exclusion of individuals with incomplete data. No patients required conversion to open surgery. This resulted in 103 in the Hugo-RARP group and 276 patients in the daVinci-RARP group. Following this, 198 patients (52.2%) were successfully matched based on their propensity scores (Figure 1). At one-year follow-up visits, data were available for all patients in the Hugo-RARP group and in the daVinci-RARP group after matching.

**Figure 2:**
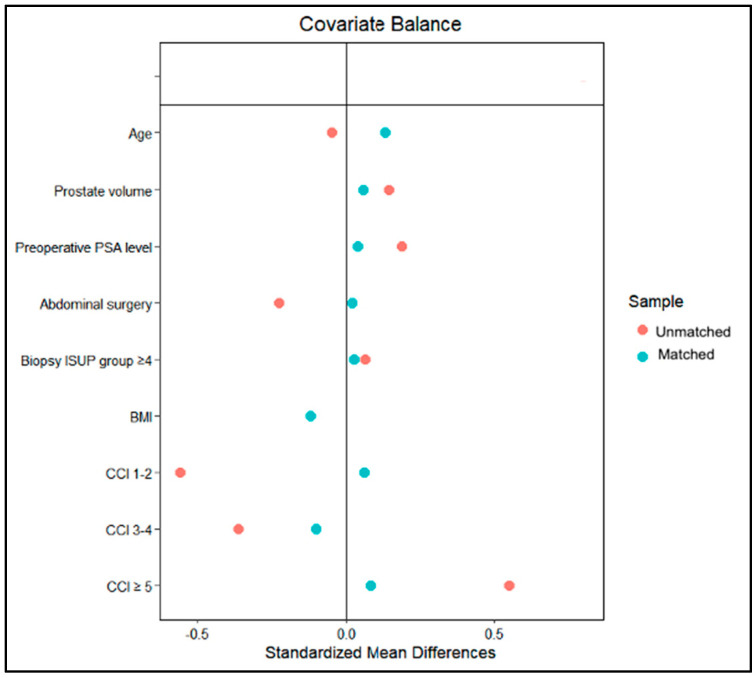
Covariate balance between Hugo and daVinci patient groups.

Upon evaluating the diagnostic graph for covariate balance (Figure 2), the propensity score matching demonstrated a satisfactory balance between the Hugo-RARP and daVinci-RARP groups, with the exception of age and BMI, which slightly exceeded the 0.1 threshold for SMD, measuring 0.136 and 0.122, respectively. Despite this, these discrepancies were deemed nonsignificant, taking into account the *p*-values.

**Figure 3:**
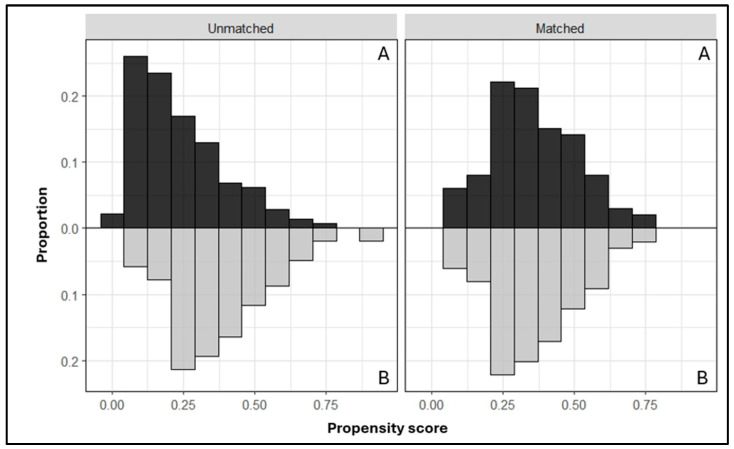
Evaluation of the common support assumption for the PS matching procedure (A = daVinci; B = Hugo).

Accordingly, a satisfactory degree of overlap in the propensity score between groups was observed (Figure 3), and the SMD in propensity score between matched subjects was 0.023.

Table 1 shows patients’ baseline characteristics in the matched population, grouped according to the surgical system employed for the procedure. In the daVinci-RARP and Hugo-RARP groups, 74 (74.7%) and 72 (72.7%) patients, respectively, had preoperative IIEF ≥ 17 scores without significant differences between the two platforms (*p* = 0.616).

### 4.2. Primary Outcome

Table 2 presents the trifecta rate one-year post-surgery, stratified by the entire cohort as well as by those who underwent nerve-sparing procedures (monolateral or bilateral) and those who did not. No statistically significant difference was observed between the two groups. The trifecta rate was 25.25% in the Hugo-RARP group and 27.27% in the daVinci-RARP group. As expected, in the nerve-sparing population, the rate for these outcomes was higher, with trifecta rates of 36.84% and 35.59% in the Hugo-RARP and daVinci-RARP groups, respectively.

### 4.3. Secondary Outcomes

Table 3 shows the functional outcomes at one-year follow-up. No significant differences were found between daVinci and Hugo™ patients in terms of social continence, with a rate of 89.9% for the daVinci-RARP group and 87.87% for the Hugo-RARP group.

In the population categorized as incontinent (10 patients in the daVinci group and 12 patients in the Hugo group), we evaluated both the 24-h pad number and the 24-h pad weighing test. The average number (±SD) of pads used in 24 h was 0.68 ± 1.08 in the daVinci-RARP group and 0.71 ± 1.02 in the Hugo-RARP group, while the average pad weight was 449 ± 497 g vs. 433 ± 508 g, respectively, with no statistically significant difference.

Data on erectile function are shown for the entire population, as well as for those who underwent monolateral or bilateral nerve-sparing procedures. Nerve-sparing surgeries were performed in 59.6% and 57.3% of daVinci-RARP and Hugo-RARP patients, respectively, without statistically significant difference (*p*-value 0.844): specifically, bilateral nerve-sparing surgeries were carried out in 34.3% and 30.3% of daVinci-RARP and Hugo-RARP patients, respectively, and unilateral nerve-sparing surgeries were carried out in 26.3% and 27.3% of daVinci-RARP and Hugo-RARP patients, respectively. Patients considered potent one-year post-surgery, with an IIEF ≥ 17, were 33.3% in the daVinci group vs. 34.3% in the Hugo group, with a higher rate for the daVinci patients, although the difference was not statistically significant (*p* = 0.881). In the subset of patients who underwent monoliteral or bilateral nerve-sparing procedures, the rates were 44.06% vs. 36.84%, respectively, with no significant difference. When patients were asked to indicate whether they had an erection hard enough for penetration, the rate observed in the entire population was 34.3% vs. 32.3% in the daVinci vs. Hugo-RARP groups, respectively, with no statistically significant difference (*p* = 0.104).

Table 4 presents the oncological outcomes at 1-year follow-up. Undetectable PSA (<0.01 ng/mL) was observed in 88.8% of daVinci patients and 92.9% of Hugo patients, with a mean (±SD) value of 0.05 ± 0.25 vs. 0. ± 0.48, respectively, with no statistically significant differences. Besides the 1-year undetectable PSA rate, Table 4 also shows detailed post-operative pathological findings in the matched population. No statistically significant differences emerged in the final pathology between the two platforms.

Table 5 presents the model analysis evaluating the association between a specific robotic platform (Hugo vs. daVinci) and functional and oncological outcomes in the propensity score-matched cohort, showing no statistically significant differences in social continence (*p* = 0.784), PSA undetectable (*p* = 0.162), and erections (*p* = 0.130)

## 5. Discussion

As far as we know, this is the first study comparing 1-year functional and oncological outcomes of RARP performed with Hugo™ RAS and daVinci surgical robotic systems using propensity-score matching (Canadian task force classification II-2) in order to perform the analysis in a quasi-randomized fashion. Previous studies have mostly focused on short-term perioperative outcomes, such as operative time and blood loss, while our study shifts attention to long-term patient-centered outcomes, such as continence, erectile function, and oncological safety. This is critical for clinical decision making and patient counseling, as it allows surgeons to provide a more holistic view of expected recovery.

Functional and oncological outcomes following radical prostatectomy play a major role in patient satisfaction, but these outcomes are not independent, meaning that some improvements in the oncological outcome may come at the expense of continence and potency. Taken by themselves, these outcome rates may not adequately inform patients of the likelihood of returning to their overall preoperative state [34]. For this reason, to compare the long-term functional and oncological outcomes of two robotic surgery platforms when performing RARP, we chose a composite outcome, such as a trifecta outcome [25], as we believe that these can effectively summarize the platform’s performance. In particular, the trifecta outcome is widely recognized for its ability to offer a comprehensive overview of surgical success. Despite its inherent complexity, it serves as a practical tool to balance the delicate interplay between oncological control and the preservation of functional abilities.

After adjusting for potential treatment selection bias by means of propensity score matching, no significant differences were found between Hugo-RARP and daVinci RARP in terms of trifecta rates at 1-year follow-up after surgery. This lack of statistically significant differences reinforces the idea that the learning curve associated with the introduction of the Hugo™ RAS platform can be rapidly overcome by experienced surgeons, allowing them to achieve comparable outcomes to those obtained with the more established daVinci system. Moreover, this supports the ongoing integration of newer robotic platforms into routine clinical practice.

Both Hugo and daVinci trifecta rates were significantly affected by potency rates, which showed an understandable improvement when considered only in the nerve-sparing subgroup, where patients achieving the trifecta were almost 10% more for both platforms.

However, no significant differences emerged between Hugo and daVinci RARP at 1-year follow-up, even considering the rate of potency recovery by itself, although a trend towards greater potency recovery at one year was observed in patients operated on the daVinci platform. Our data on erectile function may have been biased by the high mean patient age and the low median value of the preoperative IIEF-5 score. However, as known, recovery of potency may require more than twelve months [34]. Saranchuk et al., when evaluating trifecta rate after radical prostatectomy in a population with a mean patient age of 58 years, the probability of recovery of potency was 37% at 1 year (95% CI, 32 to 42) and 62% at 2 years (95% CI, 55 to 69) [35]. The most recent systematic review and metanalysis of comparative studies on early post-operative daVinci and Hugo RARP outcomes collected three studies reporting erectile function recovery, but with a maximum follow-up of three months, finding no significant differences between the two platforms at pooled analysis (OR 2.17, 95% CI 0.85–5.51; *p* = 0.103) [36]. Brime Menendez et al., at 6 months after surgery, found no significant differences in terms of mean IIEF-5 score (11.57 daVinci vs. 13.45 Hugo, *p*-value 0.36), even though they did not report a potency recovery rate [16].

Also, for the 1-year continence rate taken by itself, no significant difference emerged between the two robotic platforms. Interestingly, for patients operated with both Hugo and daVinci platforms, there was a similar improvement in the continence rate from the 3-month follow-up (73.7% vs. 74.7%; *p* = 0.35) presented in our previous study [17] to the 1-year follow-up visit (87.8% vs. 90.6%; *p* = 0.51). The above-mentioned systematic review and metanalysis, a pooled analysis of six comparative studies, found no significant differences in terms of social continence recovery at 3 months after surgery (OR 1.11, 95% CI 0.80–1.55; *p* = 0.529) [36]. Similar findings were reported by Brime Mendez et al. at 6 months after surgery (92% daVinci vs. 88% Hugo, *p*-value 0.07) [16].

As for oncological outcomes, in our previous propensity score matched comparative analysis of perioperative outcomes, we found that the rates of positive surgical margins (PSM) were comparable between Hugo-RARP and daVinci-RARP (22.2% vs. 25.2%) without statistically significant differences between the two surgical platforms (*p*-value 0.61) [17]. In particular, both Hugo-RARP and daVinci-RARP PSM rates were similar also for all the considered margin locations, as well as for the multifocal PSM rates. These results are in line with those of other non-matched series published by other authors [12,15,16,23] and two recent meta-analyses of early post-operative comparative studies [36,37].

In the present study, we also found no significant differences in terms of 1-year BCR, with comparable undetectable PSA rates between Hugo and daVinci patients (91.3% vs. 85.4%; *p* = 0.16). Our results demonstrated how the oncological safety of RARP performed with Hugo™ RAS was maintained even 1 year after surgery when compared to its reference standard, which is daVinci RARP.

Moreover, through multiple models, we were able to determine that there was no association between a particular robotic platform and significant functional and oncological outcomes, further supporting the findings from the propensity score matching analysis.

The key strengths of our study are its multi-surgeon series, and the application of the propensity score matching method, supported by multiple model analyses and sensitivity analyses. This approach ensured a comparison between groups that were uniform in terms of key potential selection biases, contributing to high-quality reporting of the results. Nonetheless, the predictive accuracy of the logistic regression model could have been affected by data imbalance, given the Hugo-RARP to daVinci-RARP ratio was 1:1 in the matched population but 1:2.6 in the un-matched population. Limitations also include this study’s retrospective design, despite being based on a prospectively collected database. Additionally, although our study is the first to compare functional and oncological outcomes of RARP performed with Hugo and daVinci at 1-year follow-up with propensity-score matching, this follow-up may not be long enough to fully evaluate these outcomes, thus making a comprehensive and definitive comparison between the two robotic surgery platforms performing RARP.

In conclusion, our study demonstrated how patients undergoing RARP with Hugo^TM^ RAS reached, at 1-year follow-up, a functional and oncological status comparable to those of patients operated with the daVinci^TM^ system by the same surgeons, proving that surgeons’ proficiency was maintained when transitioning from a well-known robotic platform to the new one. However, these findings need to be confirmed by comparative studies between larger series and with a longer follow-up.

## Figures and Tables

**Table 1:** Baseline characteristics of the patients in the matched population.

Median (1st–3rd q) or n (%)	daVinci (N = 99)	Hugo (N = 99)	*p*-Value
Age (years)	67 (62–71)	68 (62.5–72)	0.259
Prostate volume (mL)	45 (32–61)	50 (34–64.5)	0.473
Preoperative PSA level (ng/mL)	8 (5.9–11.6)	7.7 (5.4–10.0)	0.735
BMI (kg/m^2^)	27 (25–28.4)	26 (24.2–28)	0.304
Biopsy ISUP group ≥ 4	15 (15.2)	16 (16.2)	1
Abdominal surgery	37 (37.4)	38 (38.4)	1
Charlson Comorbidity Index			0.724
1–2	2 (2)	3 (3)	
3–4	45 (45.5)	40 (40.4)	
≥5	52 (52.5)	56 (56.6)	
IIEF-5 ≥ 17, n (%)	74 (74.7)	72 (72.7)	0.749
IIEF-5, median (1st–3rd q)	15 (9–20)	16 (11–21)	

**Table 2:** Trifecta rates at 1-year follow-up for the entire cohort and the nerve-sparing population.

**Entire Population**	**daVinci** **(N = 99)**	**Hugo** **(N = 99)**	***p*-Value**
Trifecta, n (%)	27 (27.27)	25 (25.25)	0.743
**Nerve-Sparing Population**	**daVinci** **(N = 59)**	**Hugo** **(N = 57)**	***p*-Value**
Trifecta, n (%)	21 (35.59)	21 (36.84)	0.889

**Table 3:** Functional outcomes at 1-year follow-up.

	daVinci (N = 99)	Hugo (N = 99)	*p*-Value
Social continence, n (%)	89 (89.9)	87 (87.9)	0.327
24 h pad number in incontinent patients			
Median (1st–3rd q)	0 (0–1)	0.5 (0–1)	0.842
Mean ± SD	0.68 ± 1.08	0.71 ± 1.02	
24 h pad weight in incontinent patients			
Median (1st–3rd q)	120 (55–875)	80 (30–875)	0.943
Mean ± SD	449 ± 497	433 ± 508	
**Potency (entire population)**			0.881
IIEF ≥ 17, n (%)	33 (33.3)	34 (34.3)	
IIEF < 17, n (%)	66 (66.7)	65 (65.7)	
Yes, hard enough for penetration	34 (34.3)	32 (32.3)	0.062
Yes, not sufficient for penetration	18 (18.2)	25 (25.3)	
No	47 (47.5)	42 (42.4)	
**Potency (nerve-sparing population)**	**daVinci** (N = 60)	**Hugo** (N = 57)	***p*-Value**
IIEF ≥ 17, n (%)	26 (44.06)	21 (36.84)	0.665
IIEF < 17, n (%)	33 (55.93)	36 (63.15)	

**Table 4:** Oncological outcomes at 1-year follow-up.

	daVinci (N = 99)	Hugo (N = 99)	*p*-Value
PSA undetectable (<0.01 ng/mL)	88 (88.8)	92 (92.9)	0.158
PSA value			
Median (1st–3rd q)	0.001 (0–0.010)	0.010 (0–0.023)	0.314
Mean ± SD	0.047 ± 0.249	0.093 ± 0.457	
Positive surgical margins ^a^	25 (25.3%)	22 (22.2%)	0.616
Pathological stage T at final histology ^a^			0.625
pT2a	2 (2.0%)	2 (2.0%)	
pT2b	1 (1.0%)	0 (0%)	
pT2c	66 (66.6%)	73 (73.7%)	
pT3a	13 (13.1%)	13 (13.1%)	
pT3b	17 (17.2%)	11 (11.1%)	
Pathological stage N at final histology ^a,§^			
pN0	28 (82.4%)	22 (91.7%)	0.143
pN1	6 (17.6%)	2 (8.3%)	

^a^ At post-operative pathological findings. ^§^ In patients who underwent pelvic lymphadenectomy.

**Table 5:** Association between the robotic surgical system (Hugo vs. daVinci) and 1-year post-operative outcomes in propensity score-matched population ^a,b^.

N = 198	OR (95%IC) HUGO (1) vs. daVinci (0)	*p*-Value
Social continence	1.129 (0.470, 2.736)	0.784
PSA undetectable	0.395 (0.155, 1.007)	0.162
Potency	1.495 (0.889, 2.524)	0.130

OR = odds ratio; CI = confidence interval. ^a^ The daVinci group was used as the reference group. ^b^ The propensity score was estimated using covariates such as age, BMI, CCI, previous abdominal surgery, preoperative PSA, prostate volume, and biopsy ISUP group.

## Data Availability

The data presented in this study are available on request from the corresponding author.

## Supplementary Materials

The following supplementary contents are available for download at https://www.mdpi.com/article/10.3390/jcm13226910/s1; Table S1: Full guidelines for reporting propensity score analysis, modified from the STROBE Statement.
